# One-year stable pilot-scale operation demonstrates high flexibility of mainstream anammox application

**DOI:** 10.1016/j.wroa.2023.100166

**Published:** 2023-01-10

**Authors:** Min Zheng, Huijuan Li, Haoran Duan, Tao Liu, Zhiyao Wang, Jing Zhao, Zhetai Hu, Shane Watts, Jia Meng, Peng Liu, Maxime Rattier, Eloise Larsen, Jianhua Guo, Jason Dwyer, Ben Van Den Akker, James Lloyd, Shihu Hu, Zhiguo Yuan

**Affiliations:** a Australian Centre for Water and Environmental Biotechnology, The University of Queensland, St Lucia, QLD 4072, Australia; b Urban Utilities, Brisbane, QLD, 4000, Australia; c South Australian Water Corporation, 250 Victoria Square, Adelaide SA 5000, Australia; d Melbourne Water, 990 La Trobe St, Docklands, VIC, 3000, Australia

**Keywords:** Mainstream anammox, Autotrophic nitrogen removal from wastewater, Bioenergy recovery, Effluent quality, NOB suppression

## Abstract

Mainstream nitrogen removal via anammox is widely recognized as a promising wastewater treatment process. However, its application is challenging at large scale due to unstable suppression of nitrite-oxidizing bacteria (NOB). In this study, a pilot-scale mainstream anammox process was implemented in an Integrated Fixed-film Activated Sludge (IFAS) configuration. Stable operation with robust NOB suppression was maintained for over one year. This was achieved through integration of three key control strategies: i) low dissolved oxygen (DO = 0.4 ± 0.2 mg O_2_/L), ii) regular free nitrous acid (FNA)-based sludge treatment, and iii) residual ammonium concentration control (NH_4_^+^ with a setpoint of ∼8 mg N/L). Activity tests and FISH demonstrated that NOB barely survived in sludge flocs and were inhibited in biofilms. Despite receiving organic-deficient wastewater from a pilot-scale High-Rate Activated Sludge (HRAS) system as the feed, the system maintained a stable effluent total nitrogen concentration mostly below 10 mg N/L, which was attributed to the successful retention of anammox bacteria. This study successfully demonstrated large-scale long-term mainstream anammox application and generated new practical knowledge for NOB control and anammox retention.

## Highlights

• Stable mainstream anammox is demonstrated at pilot scale over one year.
• High level of nitrogen removal is maintained with effluent total nitrogen < 10 mg N/L.
• NOB suppression is robust, attributed to integration of multiple control strategies.
• Anammox retained in anoxic and oxic zones jointly contribute to nitrogen removal.

## Graphical abstract

**Figure:**
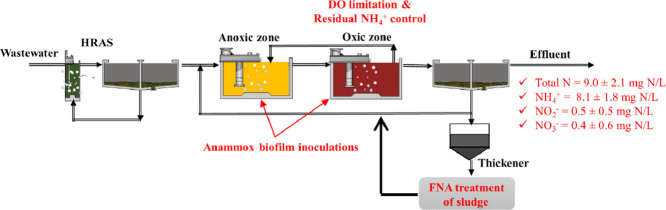

## Introduction

The microbial process of anaerobic ammonium oxidation (anammox) was discovered in late 1990s (Mulder et al., 1995), and since then researchers have proposed an innovative technology – Partial Nitritation and Anammox (PN/A) – for highly efficient nitrogen removal from wastewater, as a cost-effective alternative to the conventional nitrification and denitrification processes (Kuenen 2008). With significant efforts made, water engineers successfully installed PN/A in the sidestream line treating high-strength wastewater (Lackner et al., 2014), and are exploring PN/A in the main line of wastewater treatment plants (WWTPs). The extension to mainstream nitrogen removal can multiply the economic benefits, as the mainstream nitrogen load is about five times greater than the sidestream (Wang et al., 2022). The application of mainstream PN/A can also maximize the capture of organic carbon for bioenergy (i.e., methane) production, offsetting energy consumption in a WWTP and hence can potentially transform WWTPs from energy-consumers to energy-exporters (Kartal et al., 2010; McCarty et al., 2011). In the path to mainstream PN/A application, however, the critical challenge is the suppression of nitrite-oxidizing bacteria (NOB). NOB compete for the nitrite substrate with anammox bacteria, and its suppression is essential, but challenging under the conditions of low influent ammonium (NH_4_^+^) concentration and low operational temperature (Agrawal et al., 2018; Cao et al., 2017; Qiu et al., 2021; Wang et al., 2022).

The NOB control strategies developed to date can be divided into two major categories: *in-situ* NOB suppression and *ex-situ* NOB inactivation. The *in-situ* control comprises the use of low dissolved oxygen (DO) (Blackburne et al., 2008), real-time controlled intermittent aeration (Ma et al., 2017b; Miao et al., 2022; Regmi et al., 2014), step feed (Duan et al., 2022), short sludge retention time (SRT) (Laureni et al., 2019), residual NH_4_^+^ control (Poot et al., 2016), or creating acidic conditions (Meng et al., 2022; Wang et al., 2021b). The *ex-situ* control includes the use of harsh physical/chemical treatments such as free nitrous acid (FNA) (Wang et al., 2014), free ammonia (FA) (Wang et al., 2017), sulfide (Seuntjens et al., 2018), heat/ultrasonic shock (Chen et al., 2016), and light irradiation (Yang et al., 2022; Zheng et al., 2019), among others. It should be noted that most of these strategies were only tested in laboratories under well-controlled conditions. As the essential step prior to full-scale application, successful demonstration of mainstream PN/A process at a pilot scale remains sparse (Hausherr et al., 2022, Trojanowicz et al., 2016, Wu et al., 2021).

The level of NOB suppression is subject to the diversity of NOB members, across four genera *Nitrobacter, Nitrospira, Candidatus* (*Ca.*) Nitrotoga, and *Nitrolancea* that are commonly found in wastewater treatment systems (Daims et al., 2016). The NOB invasion from wastewater (Duan et al., 2019b) further adds to the complexity, meaning that some NOB genera or species can adapt to the aforementioned control strategies, leading to failure of NOB suppression during a long-term operation (Duan et al., 2019a; Liu and Wang 2013; Wang et al., 2021a). For example, *Ca.* Nitrotoga fabula, a newly isolated NOB from an activated sludge sample (Kitzinger et al., 2018), was found to possess strong resistance to ex-situ exposure of FNA above one parts per million (> 1 mg HNO_2__—_N/L) (Zheng et al., 2020). These recent studies illustrate the importance of suppressing diverse NOB through the integration of multiple strategies. Therefore, this study aims to develop a combination of operational strategies to suppress the growth of NOB in a mainstream pilot system. Over one year of operation, this system successfully demonstrated the robustness of the mainstream PN/A process, thus opening a path to full-scale implementation.

## Results

### Long-term system operation and performance

The pilot system comprised an integrated fixed film activated sludge (IFAS) process with the classical continuous-flow anoxic (A) and oxic (O) configuration and inoculation of anammox-contained carriers in both the A and O zones (Fig. S1). The system was fed with domestic wastewater pre-treated with a High Rate Activated Sludge (HRAS) process, which usually captured ∼60% of chemical oxygen demand (COD) from raw sewage (Table S1). The designed SRT of 12 d and hydraulic retention time (HRT) of 3.2 h in the A zone and 6.7 h in the O zone are representative parameters of the conventional activated sludge processes installed across the globe (Rittmann and McCarty 2001). Two NOB control strategies of low DO at 0.4 ± 0.2 mg O_2_/L in the O zone and external sludge treatment using FNA at ∼2 mg HNO_2__—_N/L, which were optimized in our previous laboratory study (Wang et al., 2016), were initially integrated in the pilot system.

After start-up, the system successfully retained anammox bacteria in both the A- and O-biofilms (Fig. S2a). The TN and NH_4_^+^ removal efficiencies gradually increased to over 80% in two months (Fig. S2b, S2c). We monitored the maximum activity of AOB and NOB in sludge flocs using *ex-situ* batch tests on a weekly basis, which showed that at around 100th day, the NOB activity unexpectedly increased and reached 3 mg NO_2_^−^-N/(g volatile suspended solids (VSS)·h) in two weeks (Fig. S2d). This posed a risk of failed NOB control, and thus immediately, the *in-situ* DO setpoint was lowered to ∼0.2 mg O_2_/L. As expected, the NOB activity of sludge flocs decreased rapidly to below 0.2 mg NO_2_^−^-N/(g VSS·h) following this change. However, this also caused a significant deterioration in the NH_4_^+^ removal efficiency, reaching as low as ∼30%, indicating that the AOB activity was also negatively affected by the low DO concentration. Consequently, the DO setpoint was elevated back to 0.4 mg O_2_/L, and a residual NH_4_^+^ concentration control (∼8 mg N/L) was implemented. Maintaining a residual NH_4_^+^ level was hypothesized to suppress NOB activity by promoting anammox activity for nitrite competition and decreasing oxygen penetration in biofilms, which will be further elaborated in section 2.4.

**Fig. 1:**
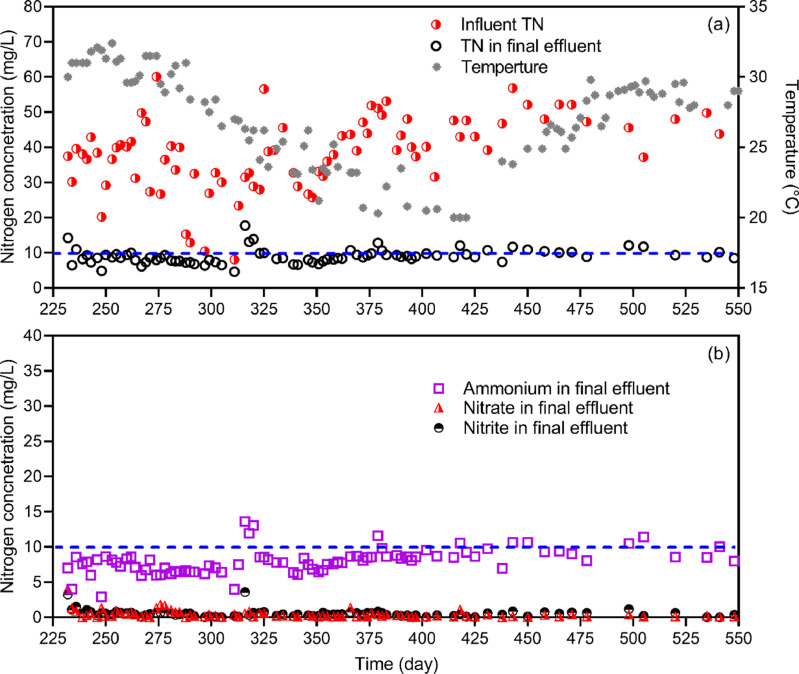
Profiles of influent and effluent TN concentrations, temperature (a) and ammonium, nitrite and nitrate nitrogen concentrations in the final effluent (b) throughout the one-year stable operation of the pilot A/O system. The dashed line represents a nitrogen concentration of 10 mg N/L.

The improved operational strategy consisted of three key controls: the external FNA sludge treatment, *in-situ* low DO concentration (0.4 ± 0.2 mg O_2_/L), and a relatively high residual NH_4_^+^ concentration (∼8 mg N/L). With a fluctuating TN concentration in real wastewater and seasonally varying temperature, the system effluent had mostly maintained a TN concentration below 10 mg N/L, comprising of NH_4_^+^ (∼8 mg N/L), NO_2_^−^ (∼1 mg N/L) and NO_3_^−^ (∼1 mg N/L) (Fig. 1). This resulted in averaged TN and NH_4_^+^ removal efficiencies of > 80% over a one-year period, and therefore demonstrated a successful and robust operation of efficient mainstream nitrogen removal process.

### Retention of anammox bacteria in the A- and O-biofilms

The efficient nitrogen removal performance that was achieved during the pilot trial was largely attributed to anammox, because the HRAS pretreated wastewater only supported limited nitrate- and nitrite-dependent heterotrophic denitrification rates, measured at 0.8 and 1.5 mg N/(g VSS·h), respectively. These rates are comparable to the sludge denitrification rates in the absence of soluble carbon sources (e.g., 0.7–1.4 mg NO_3_^−^-N/(g VSS·h) (Zheng et al., 2013)), and were much lower than the denitrification rate measured in domestic wastewater with readily biodegradable organic matters (e.g., 6.6 mg NO_3_^−^-N/(g VSS·h) (Kujawa and Klapwijk 1999)). The low organic degradability in wastewater with HRAS pretreatment was also reflected by a low TN removal efficiency of only 10.4% ± 4.6% in our laboratory control reactor that used the same influent as the pilot system (Wang et al., 2021a).

**Fig. 2:**
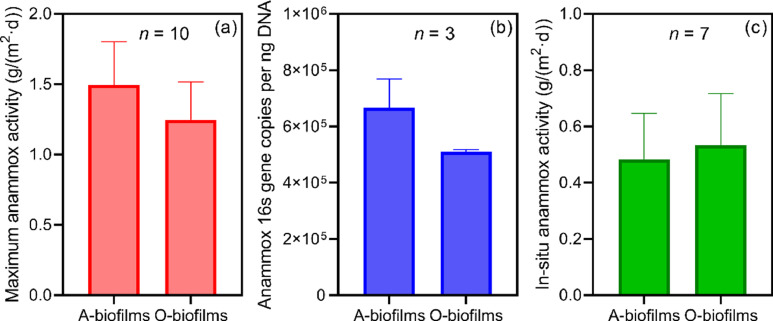
Maximum activities (a), abundance (b), and in-situ activities (c) of anammox bacteria in the A- and O-biofilms during the stable operation of the pilot system.

The 16S rRNA gene amplicon sequencing analysis revealed that anammox bacteria dominated in the A- and O-biofilms were *Candidatus* Brocadia, showing no difference at the genus level (Fig. S3). The abundance of *Candidatus* Brocadia within the A-biofilms was relatively higher at 0.74% ± 0.21% compared to the O-biofilms (0.30% ± 0.19%), suggesting a slightly higher capacity of A-biofilms to retain anammox bacteria. The use of *ex-situ* batch tests and q-PCR analyses also showed that the maximum activity and population of anammox bacteria within the A-biofilms were 1.5 ± 0.3 g N/(m^2^-carrier·d) and 6.6 ± 1.0 × 10^5^ 16S gene copies per ng DNA, respectively, which were comparable but slightly higher than those measured with the O-biofilms (Fig. 2a-2b). These differences in anammox abundance and activity may be related to differences in environmental conditions such as DO, nitrite concentration and shear force between the A and O zones. Together, these results indicate the successful retention of anammox bacteria in both the anoxic and oxic tanks, thus expanding the application of mainstream anammox technology.

**Fig. 3:**
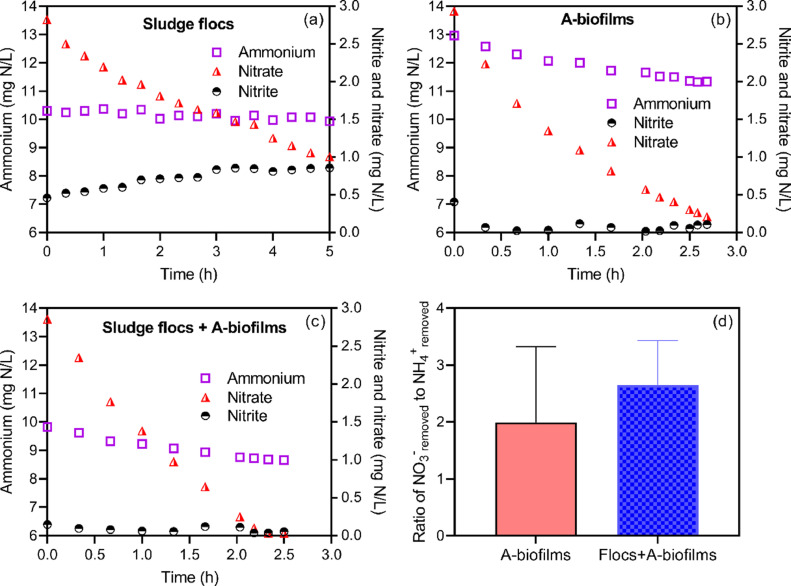
Profiles of ammonium, nitrite and nitrate in anoxic batch tests using sludge flocs (a), A-biofilms (b) and combined biofilms and flocs (c). (d) The ratios of NO_3_^−^ removed to NH_4_^+^ removed calculated in the tests of (b) and (c).

Some studies have recently revealed that the A-biofilms are the ideal location for retaining anammox bacteria in a continuous-flow nitrogen removal process, as anoxic environments can promote anammox enrichment which obtain nitrite from partial denitrification (Li et al., 2021; Li et al., 2019). Herein, we examined whether nitrate-dependent denitrification with HRAS effluent could support the A-biofilm anammox, i.e., partial denitrification and anammox (PD/A). The removal of NH_4_^+^ and NO_3_^−^ without NO_2_^−^ supply was tested in a series of anoxic batch assays. NH_4_^+^ and NO_3_^−^ simultaneously decreased in the groups with the A-biofilms, while in the group without A-biofilms (i.e., sole sludge flocs), only the NO_3_^−^ concentration decreased (Fig. 3a-c). This suggests that the NH_4_^+^ removal in the anoxic tank was driven by anammox bacteria in the A-biofilms, which obtained NO_2_^−^ from partial denitrification occurring in both flocs and biofilms. The molar ratios of NO_3_^−^ removed to NH_4_^+^ removed in batch tests with A-biofilms were calculated to be 2.0–2.6 on average (Fig. 3d). This suggests that more than 50% of NO_3_^−^ could be partially reduced to NO_2_^−^ and supplied to the anammox bacteria.

### Contribution of anammox to nitrogen removal

We developed a kinetic method to estimate the *in-situ* anammox activity based on the measured maximum rate multiplied by a Monod equation incorporating substrates and inhibitors, as described in the Activated Sludge Models (Henze et al., 2000). This was because NO_2_^−^, with measured concentrations averaging at 0.24 mg N/L in the A zone and 0.53 mg N/L in the O zone (Fig. S4), were comparable to the apparent nitrite affinity constant of 0.42 mg NO_2_^−^-N/L for the A-biofilm and 0.38 mg NO_2_^−^-N/L for the O-biofilm (Fig. S5). Thus, the *in-situ* anammox activity was limited by the *in-situ* nitrite concentration.

To this end, short-term batch tests were first carried out to measure the maximum anammox activity in the A- and O-biofilms in conditions with sufficient nitrite (Fig. S5). Together with the measured apparent nitrite affinity constant and the *in-situ* nitrite concentration, the *in-situ* anammox activity of the A-biofilms was estimated to be 0.48 ± 0.16 g N/(m^2^-carrier·d), comparable to that of the O-biofilm (0.53 ± 0.18 g N/(m^2^-carrier·d)) (Fig. 2c). The *in-situ* anammox activities, together with the HRT and carrier filling ratios applied to the A and O zones of the pilot system, revealed that anammox in the O- and A-biofilms contributed 60% and 40% of total nitrogen removal via anammox, respectively. Of note, the relative contribution may be influenced by the availability of organic carbon in the HRAS effluent, offering a certain level of flexibility to maintain the overall stable nitrogen removal, i.e., the A-biofilms should contribute more if more organic carbon is available and vice versa.

While the PN/A process contributed to the majority of nitrogen removal, a key role of the PD/A process is to remove NO_3_^−^ generated by anammox bacteria and the O-biofilm NOB (see details in Section 2.4). This is evidenced by the extremely low residual NO_3_^−^ concentration (< 1 mg N/L) in the final effluent, and it appeared that the denitrification process based on limited organic carbon in the HRAS effluent was adequate to consume almost all NO_3_^−^ in the pilot system. Therefore, partial denitrification also played a critical role in achieving the high-level TN removal.

### Mechanisms of stable NOB suppression

**Fig. 4:**
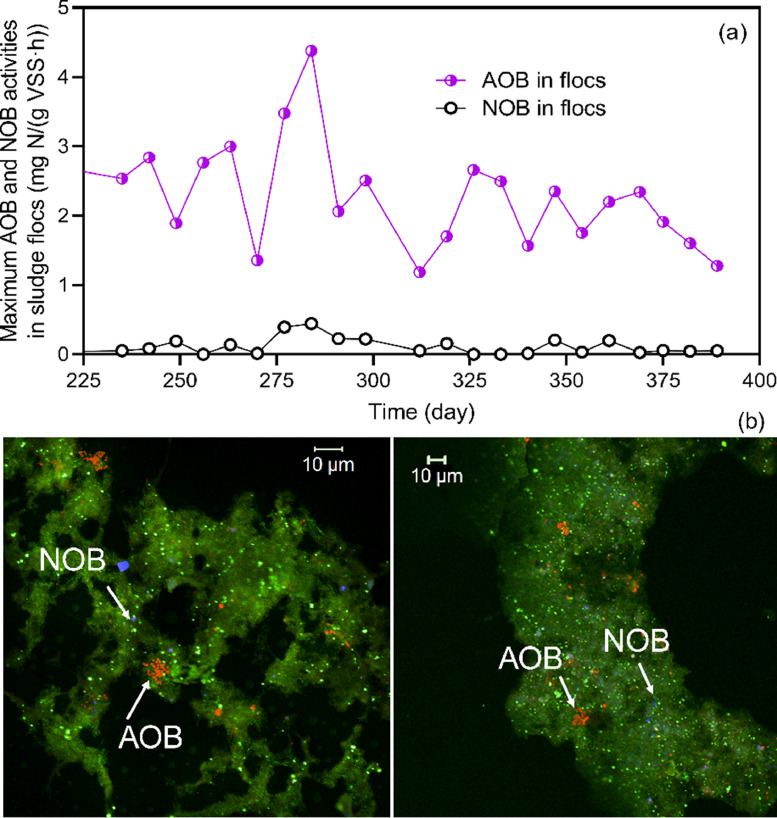
(a) Measured maximum AOB and NOB activities in sludge flocs using ex-situ batch tests under non-limited substrate conditions. (b) Representative FISH images showing significant dominance of AOB over NOB in sludge flocs. EUB mix counterstaining is in green, probes specific for Betaproteobacterial AOB (Nso1225) in red), *Nitrobacter* (Nit3) in blue) and *Nitrospira* (Ntspa662 and Ntspa712) in blue).

Restricting NOB activity in the O zone enabled the NO_2_^−^ produced by AOB to be supplied to anammox bacteria, which represent the typical PN/A pathways for nitrogen removal. NOB in sludge flocs were satisfactorily washed out in the pilot system where the maximum NOB activity in sludge flocs was only 0.1 ± 0.1 mg NO_2_^−^-N/(gVSS·h), compared to 2.3 ± 0.8 mg NH_4_^+^-N/(gVSS·h) for AOB (Fig. 4a). This was corroborated by the Fluorescence in situ hybridization (FISH) analysis, which showed that AOB considerably outnumbered NOB (Fig. 4b). The strong suppression of NOB can be largely attributed to regular sludge treatment using FNA. Taking the inoculated sludge flocs as an example, the maximum NOB activity substantially decreased from 1.2 mg NO_2_^−^-N/(gVSS·h) to less than 0.1 mg NO_2_^−^-N/(gVSS·h) after 24-h of FNA treatment. Nevertheless, it should be noted that some NOB could adapt to the sole FNA treatment (Duan et al., 2019a; Ma et al., 2017a; Zheng et al., 2020). This indicated that the use of *in-situ* low DO conditions and the nitrite competition by anammox bacteria were also critical for the long term suppression of NOB (Wang et al., 2021a).

**Fig. 5:**
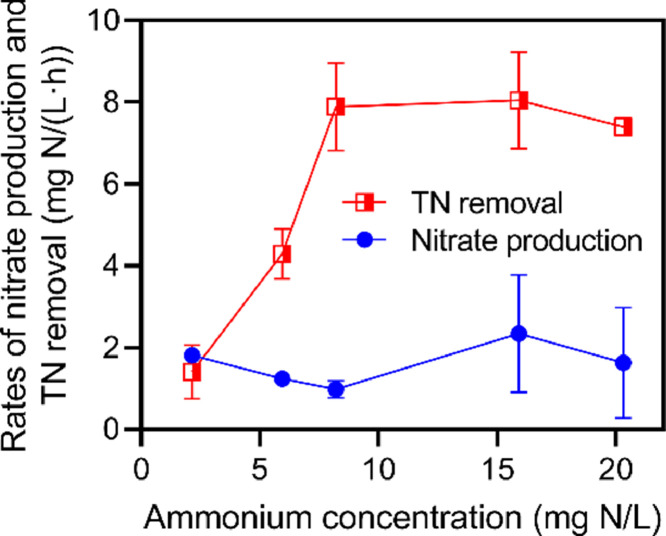
Measured TN removal and nitrate production rates of the O-biofilms under five different residual ammonium concentrations using ex-situ batch tests. The DO concentration was fixed at 0.4 mg O_2_/L during the tests.

In contrast to flocs, the O-biofilms contained NOB (*Nitrospira*) according to the 16S rRNA gene amplicon sequencing analysis (Fig. S3b). The presence and abundance of NOB in the O-biofilms were also reflected by a maximum NOB activity to maximum AOB activity ratio of about 1 (Fig. S6), which is 2–3 times higher than that for biofilms from our laboratory PN/A system reported with stable NOB suppression (Meng et al., 2021). However, under the *in-situ* conditions of low DO and relatively high residual NH_4_^+^, the ratio of NO_3_^−^ production to TN removal by the O-biofilms was only ∼20% (Fig. 5). This ratio was slightly higher than the stoichiometry of anammox reaction (i.e., 13%) and close to the ratios observed in previous laboratory PN/A systems (Laureni et al., 2016; Meng et al., 2021). This result indicated the suppression of the NOB activity in the O-biofilms by *in-situ* factors, rather than NOB elimination.

Indeed, the NO_3_^−^ production rate by the O-biofilms was measured as ∼2 mg NO_3_^−^-N/(L·h) when DO was controlled at 0.4 mg/L (Fig. 5). This was significantly lower than the maximum NOB activity rate of 4.7 mg NO_2_^−^-N/(L·h) measured at a high DO (> 8 mg O_2_/L) (Fig. S6), demonstrating the role of low DO in the suppression of the *in-situ* NOB activity. In PN/A biofilms, NOB are predominantly found within deep layers of the O-biofilms together with anammox bacteria (Zhao et al., 2023). Thus, the NOB and anammox activity should be essentially controlled by oxygen penetrating into biofilms, which could be lower than the monitored DO concentrations within the bulk liquid. Oxygen penetration in biofilms is affected by the AOB activity, meaning that the residual NH_4_^+^ concentration, which controls AOB activity, should be another important factor driving NOB suppression and anammox activity (Wang et al., 2022). By measuring the TN removal rates under different residual NH_4_^+^ concentrations in batch tests at DO of 0.4 mg O_2_/L, we found that a residual NH_4_^+^ higher than 8 mg N/L was essential to achieving a high TN removal rate by the O-biofilms (Fig. 5). In contrast, the decrease in residual NH_4_^+^ concentrations to ∼2 mg N/L dramatically reduced the nitrogen removal performance, which highlights the sensitivity of anammox activity to the residual NH_4_^+^ concentration. This result provides critical evidence to support a setpoint of residual NH_4_^+^ as high as ∼8 mg N/L in suppressing O-biofilm NOB for achieving efficient nitrogen removal in the pilot system.

## Discussion

This study successfully demonstrated stable and long-term application of anammox bacteria for mainstream nitrogen removal at pilot scale. Efficient nitrogen removal (with the effluent TN less than 10 mg N/L), was achieved using industrially relevant operating conditions over one year, demonstrating the potential for real-world application of the mainstream anammox process. A high-level contribution of anammox to mainstream nitrogen removal was achieved by integrating multiple control strategies, which included *in-situ* low DO control together with regular FNA sludge treatment for effective elimination of NOB in sludge flocs, and low DO with residual NH_4_^+^ control for suppression of NOB activity in biofilms. These results demonstrated that multiple strategies were essential to overcome critical issues that result in NOB adaptation which has previously been documented in laboratory studies (Duan et al., 2019a; Wang et al., 2021a). The pilot system also employed the HRAS process to harvest organic carbon from wastewater to maximize bioenergy recovery in support of the ongoing paradigm shift for municipal WWTPs to maximize energy recovery from wastewater.

The pilot-scale demonstration of the mainstream PN/A process has been reported in a few studies (Table S2), but not all were successful. A general challenge mentioned by most previous studies is the NOB control, leading to a relatively poor effluent quality despite achieving comparable nitrogen removal rates. For example, a nitrogen removal rate of ∼0.2 kg N/m^3^/d was achieved in a pilot PN/A system with granular sludge, whereas the total nitrogen in the effluent was mostly above 15 mg N/L (Lotti et al., 2015). Likewise, another pilot trial that employed anammox carriers for mainstream wastewater treatment was also limited by high effluent total nitrogen concentration greater than 20 mg N/L, despite achieving a peak nitrogen removal rate of 0.13 kg N/m^3^/d (Gustavsson et al., 2020). A notable exemption was Hausherr et al. (2022), where the total nitrogen was maintained below 3 mg N/L in a two-stage PN/A system. To the best of our knowledge, this is the only pilot-scale trial employing a two-stage configuration. The researchers argued that the two-stage configuration is preferable if a low effluent TN is required. In comparison, a relatively high NH_4_^+^ concentration is usually needed in an one-stage configuration, to support anammox activity and inhibit NOB activity (Wang et al., 2022), which is also demonstrated in our study. In comparison to previous trials, the extremely low nitrate in the final effluent of our system should be highlighted, which was attributed to the denitrification/partial denitrification in the anoxic tank, as well as the stable NOB elimination in flocs and NOB activity suppression in biofilms in the oxic tank.

Despite the benefits given by the residual ammonium control strategy, the effluent of the proposed mainstream anammox process needs polishing before discharging into natural water bodies as it contains NH_4_^+^ of ∼8 mg N/L. The residual NH_4_^+^ control was thought to be an important strategy for NOB suppression in mainstream PN/A processes (Laureni et al., 2016; Yang et al., 2023), while a minimal NH_4_^+^ set-point remains elusive. A model-based study suggested a residual NH_4_^+^ concentration of ∼1 mg N/L for maintaining NOB repression in oxygen-limited PN/A (Pérez et al., 2014), yet our study indicated that a higher residual NH_4_^+^ level was required.

## Conclusions

This study demonstrated the robustness of mainstream anammox technology at pilot scale. The main conclusions from this study were:

• Integration of three control strategies, including low DO, FNA sludge treatment and residual NH_4_^+^ control, was effective in the elimination of NOB in sludge flocs and suppression of NOB in biofilms;
• Effluent quality of the mainstream anammox process was satisfactory in maintaining TN concentration generally less than 10 mg N/L, while a polishing process to remove the residual NH_4_^+^ of ∼8 mg N/L would be required in practice;
• Both carriers in anoxic and oxic zones were effective in retaining anammox activity, with comparable treatment capacity and contribution to nitrogen removal.

## Material and methods

### Pilot system setup, operation, and monitoring

The pilot-scale treatment system consisted of an HRAS process for capturing organic carbon from wastewater followed by a continuous-flow A/O process for nitrogen removal (Fig. S1). The whole system was installed at the Innovation center, located in the Luggage Point Sewage Treatment Plant, Brisbane, Australia. The system was operated for one and a half years and continuously fed with screened real domestic wastewater. The HRAS system had a working volume of 0.25 m^3^, with a set HRT of 1.4 h. The HRAS was connected to a clarifier, where the HRAS sludge settled and was wasted or returned the HRAS reactor. The effluent was fed to the A/O system (0.47 m^3^ in the A zone and 0.98 m^3^ in the O zone). The anammox-containing K5 carriers, collected from a 5-m^3^ moving bed biofilm reactor (MBBR) that treated real anaerobic digestion liquor via the PN/A pathway, were added to the A and O zones with volumetric filling ratios of 48% and 33%, respectively. The HRAS pre-treated wastewater was pumped (Mono, CP11) into the A zone at a flow rate of 3.5 m^3^/d, resulting in HRT of 3.2 h in the A zone and 6.7 h in the O zone. The mixed liquor recirculation rate from the O to the A zone was set at ∼7 times the influent flow rate, i.e., 24.7 m^3^/d. The A and O zones were mixed by coarse bubbling at an air flow rate of 200 L/day. pH in the system was monitored by using a pH probe (miniCHEM, Labtek) and a transmitter (multiparameter transmitter M800, Mettler Toledo), but not controlled. DO concentration in the O zone was controlled between 0.2 and 0.8 mg O_2_/L (0.4 ± 0.2 mg O_2_/L on average) by using an on/off control of micro-bubbled air supply pump. An online NH_4_^+^ probe was installed in the O zone, which also controlled the air supply, i.e., the aeration pump was switched off when a residual NH_4_^+^ concentration was lower than a set level (i.e., 8 mg NH_4_^+^-N/L from day 225) and vice versa. These controls were merged into a programmable logic controller (PLC). SRT of the A/O system was 12 d by semi-continuously discharging mixed liquor from the O zone. Following the A/O system, a secondary settler was established to retain biomass and return it to the A zone with a sludge return rate of 3.5 m^3^/d. Both the HRAS and A/O systems were inoculated with conventional activated sludge from the full-scale Luggage Point plant.

An external unit was set up in the sidestream line of the A/O system to implement treatment of sludge from the A/O system with FNA. Activated sludge of 400 L was collected daily from the O zone and subsequently concentrated to 4 − 5 g total suspended solids (TSS)/L by using a centrifuge at 1000 rpm for 5 min. Afterwards, the thickened sludge was transferred to a 100 L treatment tank and exposed to ∼2 mg HNO_2__—_N/L (i.e., pH = 5.6 − 5.8, NO_2_^−^ = 500 mg N/L, Temperature = 25 °C). After ∼24 h exposure, the treated sludge was returned to the A zone.

The influent and HRAS effluent COD concentrations were measured 2 − 3 times per week. NH_4_^+^-N, NO_2_^−^-N, NO_3_^−^-N and PO_4_^3−^-P concentrations in the influent and effluent of the A/O system were also measured 2 − 3 times per week. The maximum anammox activity of the A- and O-biofilms, and the maximum AOB and NOB activities of the floccular sludge were analysed weekly. The mixed liquor suspended solids (MLSS) and VSS concentrations of the sludge were monitored once per week. Other chemical and microbial analyses, as well as batch tests, were carried out when the whole system reached stable operation, as detailed in 5.5.

### Chemical analysis

Concentrations of MLSS, VSS and COD were measured according to the standard methods (APHA 2005). Mixed liquor samples were filtered through 0.45 μm Millipore filters for the determination of NH_4_^+^-N, NO_2_^−^-N, NO_3_^−^-N and PO_4_^3−^-P concentrations with a Lachat QuikChem8000 Flow Injection Analyzer (Lachat Instrument, USA).

### Microbial community analysis

On day 450, floccular sludge, and the A- and O-biofilms were collected in triplicate and submitted to Australian center for Ecogenomics at The University of Queensland (https://ecogenomic.org/) for the analysis of microbial communities. DNA of the collected samples was extracted from 50 to 200 mg of each sample using Qiagen DNeasy Powersoil Pro-Kit (cat #7016) according to the manufacturer's protocol, and its quality was checked with gel electrophoresis. The 16S rRNA genes encompassing the V6 to V8 regions were targeted using the 926F (5′- AAA CTY AAA KGA ATT GRC GG −3′) and 1392wR (5′- ACG GGC GGT GWG TRC −3′) primers modified to contain Illumina specific adapter sequence (926F: 5′- TCG GCA GCG TCA GAT GTG TAT AAG AGA CAG AAA CTY AAA KGA ATT GRC GG −3′ and 1392wR: 5′- GTC TCG TGG GCT CGG AGA TGT GTA TAA GAG ACA GAC GGG CGG TGW GTR C −3′). The universal primer pair 926F-1392wR amplifies the small submit (SSU) ribosomal RNA of eukaryotes (18S) and prokaryotes (16S) specifically the V6, V7 and V8 regions. Raw sequencing data was processed by Quantitative Insights Microbial Ecology Ⅱ (QIIME Ⅱ) in multiple steps, including poor-sequences removal. The sequences were clustered into operational taxonomic units (OTUs) at 97% identify threshold.

### qPCR and FISH analyses

Real-time qPCR was conducted to quantify anammox 16S rRNA genes in the A- and O-biofilms. The qPCR amplification reaction was performed with 25 µL solution, consisting of 1 µL (10−20 ng/µL) DNA, 12.5 µL Platinum Green Hot Start PCR Master Mix (2X, ThermoFisher Scientific), 10.5 µL nuclease-free water, and 1 µL primers (20 µM), in an Applied Biosystems Veriti™ 96-Well Thermal Cycler (Model 9902). The used primer set was Amx694F (5′−3′ GGGGAGAGTGGAACTTCGG)/Amx960R (5′−3′ GCTCGCACAAGCGGTGGAGC), developed in literature (Ni et al., 2010). The thermal profile was 95 °C for 3 min × 1 cycle, 95 °C for 30 s, 56 °C for 30 s, and then 72 °C for 40 *s*× 35 cycles. The amplification efficiency was estimated to be 105.54%.

FISH analysis was carried out to verify the presence of AOB and NOB in sludge flocs. The sludge samples were fixed in 4% paraformaldehyde stock solution and then hybridized with designed oligonucleotide probes, including EUB mix (338, 338II, and 338III), Nso1225, Nit3, Ntspa662 and Ntspa712. The detailed probes, hybridization, and visualization can be found in our previous reports (Meng et al., 2021; Zheng et al., 2021).

### Monitoring of maximal activity for AOB, NOB and anammox bacteria in long-term operation

Carriers in the A and O zones (random collection of 130 pieces) and sludge flocs (0.5 L) were collected and transferred to batch reactors for the analysis of maximum anammox and AOB/NOB activities, respectively. In brief, all assays were conducted in 1-L glass bottle. Initially, NH_4_Cl and NaNO_2_ stock solutions were added to increase NH_4_^+^-N and NO_2_^−^-N concentrations to about 20–30 mg N/L, ensuring that these substrates were not rate-limiting. A magnetic stirrer was used to mix with a speed of 200 rpm. Each assay lasted for ∼3 h at room temperature (22 ± 1 °C), during which pH was maintained between 7.0 and 7.5 by manually adding 0.1 M HCL or 0.1 M NaOH. To measure the maximum anammox activity, compressed pure dinitrogen (N_2_) gas was continually flushed at 1.0 L/min. To estimate the maximum AOB and NOB activities, air was supplied at 1.0 L/min. Liquid samples were taken every 0.5 h and filtered with 0.45 μm disposable sterile Millipore filters (Merck) for the measurement of NH_4_^+^-N, NO_2_^−^-N and NO_3_^−^-N concentrations. The maximum anammox activity was determined by dividing the volumetric TN reduction rate (linear regression of TN versus time) to the K5 carrier biofilm surface area (800 m^2^/m^3^-packed volume). The maximum AOB and NOB activities were calculated to be slopes of NH_4_^+^ reduction and NO_3_^−^ production versus time divided by the VSS concentration, respectively.

### Experimental design of other ex-situ batch tests

More ex-situ batch tests were conducted in the same experimental set-up as that for the measurement of maximum activity (section 5.5), while the experimental conditions were designed according to the actual in-situ environments.

*In-situ anammox activity of the A- and O-biofilms.* The tests were performed with the initial NH_4_^+^ concentration of ∼10 mg N/L, close to that in the A and O zones. Tests using the O-biofilms were carried out at controlled DO concentration of ∼0.4 mg O_2_/L, the same as that in the O zone of the pilot A/O system. In each test, the initial NO_2_^−^ concentration was raised to ∼2 mg N/L via adding a nitrite stock solution. The test was carried out until the NO_2_^−^ concentration decreased to zero, and the liquid samples were taken every 5 min for 1–2 h. After that, the NO_2_ concentration was raised to 15 mg N/L via re-addition of the nitrite stock solution, and the experiments was continued for a further 1 h, with the liquor samples collected every 15 min. The two tests enabled the calculation of the TN removal rate (i.e., *r*) under varied NO_2_^−^ concentrations (i.e., *S*) from 0 to ∼5 mg N/L and the maximum TN removal rate (i.e., *r*_max_) separately. The data was fitted into the Monod equation (*r* = *r*_max_·(*S*/(*K* + *S*)). Through a non-linear regression of *r* versus *S*, the apparent *K*-values with respect to NO_2_^−^ of anammox in the A- and O-biofilms were obtained. Then, the in-situ anammox activity was calculated with the measured maximum anammox activity (see section 5.5), the apparent *K*-value, and the in-situ NO_2_^−^ concentrations in the A zone by using the Monod equation. For the O-biofilms, the inhibition of DO should also be considered for the calculation of in-situ anammox activity. This factor was normalized to be 0.74 in this work according to a ratio of the measured maximum anammox activity at DO of 0.4 mg O_2_/L to that at DO of 0 mg O_2_/L.

*Effect of residual* NH_4_^+^ *concentration on TN removal and* NO_3_^−^ *production rates of the O-biofilms*. This group of tests were carried out at DO of 0.4 mg O_2_/L under different residual NH_4_^+^ concentrations using the O-biofilms. Initially, 25 mg N/L of NO_2_^−^ was added to ensure the non-limited NO_2_^−^ condition.

*Simultaneous removal of* NH_4_^+^and NO_3_^−^ *in the A zone***.** To examine the anammox activity supported by partial denitrification in the A zone, three batch tests were carried out with sludge flocs, A-biofilms, and their combination. Initially, NH_4_^+^ of ∼10 mg N/L and NO_3_^−^ of ∼3 mg N/L were provided as substrates without NO_2_^−^ addition. The HRAS effluent (volumetric ratio of 1:3) was also added to support denitrification with the limited organic carbon. The operational conditions including the provision of compressed dinitrogen gas, magnetic mixing, pH control, liquor sampling and analysis were identical to those described in section 5.5.

## Declaration of Competing Interest

The authors declare that they have no known competing financial interests or personal relationships that could have appeared to influence the work reported in this paper.

## Data Availability

Data will be made available on request

## Appendix Supplementary materials

Image, application 1
