# Supplementary material for: One-year stable pilot-scale operation demonstrates high flexibility of mainstream anammox application
Source: Water Res X. 2023 Jan 10;19:100166. doi: 10.1016/j.wroa.2023.100166 (PMC9845764; doi:10.1016/j.wroa.2023.100166)
Supplement: Supplementary file 1 [file mmc1.docx]

**Supplementary materials**

**One-year stable pilot-scale operation demonstrates high flexibility of mainstream anammox application**

Min Zheng,^a^ Huijuan Li,^a,b^ Haoran Duan,^a^ Tao Liu,^a^ Zhiyao Wang,^a^ Jing Zhao,^a^ Zhetai Hu,^a^ Shane Watts,^a^ Jia Meng,^a^ Peng Liu,^a^ Maxime Rattier,^a^ Eloise Larsen,^a^ Jianhua Guo,^a^ Jason Dwyer,^b^ Ben Van Den Akker,^c^ James Lloyd,^d^ Shihu Hu,^a,*^ Zhiguo Yuan^a,*^

^a^ Australian Centre for Water and Environmental Biotechnology, The University of Queensland, St Lucia, QLD 4072, Australia

^b^ Urban Utilities, Brisbane, QLD, 4000, Australia

^c^ South Australian Water Corporation, 250 Victoria Square, Adelaide SA 5000, Australia

^d^ Melbourne Water, 990 La Trobe St, Docklands VIC 3000, Australia

**Corresponding authors**

Email addresses: [z.yuan@uq.edu.au](mailto:z.yuan@uq.edu.au) (Zhiguo Yuan); [s.hu@uq.edu.au](mailto:s.hu@uq.edu.au) (Shihu Hu)

Number of pages: 4

Number of tables: 2

Number of figures: 6

**Table S1**. Characteristics of domestic wastewater before and after pretreatment by the HRAS process. The HRAS effluent was then used as influent of the pilot nitrogen removal system (average value ± standard deviation).

| Parameters | Raw domestic wastewater (before HRAS) | Pretreated wastewater (after HRAS) |
| --- | --- | --- |
| NH_4_^+^-N (mg/L) | 46 ± 14 | 44 ± 12 |
| NO_2_^-^-N (mg/L) | 0.1 ± 0.1 | 0.1 ± 0.1 |
| NO_3_^-^-N (mg/L) | 0.1 ± 0.1 | 0.1 ± 0.1 |
| Total suspended solids (TSS, mg/L) | 559 ± 321 | 203 ± 270 |
| Volatile suspended solids (VSS, mg /L) | 459 ± 231 | 145 ± 48 |
| Total COD (TCOD, mg/L) | 910 ± 286 | 359 ± 86 |
| Soluble COD (SCOD, mg/L) | 283 ± 76 | 151 ± 37 |
| PO_4_^3−^-P (mg/L) | 5 ± 1 | 5 ± 1 |
| pH | 7.1 - 7.3 | 7.1 - 7.3 |

**Table S2**. Comparisons between pilot scale trials of previous studies and this work.

| Regions | Configuration | Strategies for NOB control | Effluent quality | TN removal efficiency | Reference |
| --- | --- | --- | --- | --- | --- |
| Australia | Conventional A/O process with anammox carriers in both A and O zones | DO set point: 0.4 mg O_2_/L  Residual NH_4_^+^ set point: 8 mg N/L  Regular FNA treatment | ~8 mg NH_4_^+^-N/L,  ~1 mg NO_2_^-^-N/L,  ~1 mg NO_3_^-^-N/L | ~80% | This work |
| Netherlands | One-stage with anammox granules | DO set point: 0–2.0 mg O_2_/L | 6.8 mg NH_4_^+^-N/L,  2.3 mg NO_2_^-^-N/L,  9.1 mg NO_3_^-^-N/L | < 50% | Lotti et al. 2015 |
| Sweden | One-stage with anammox carriers | DO: 1.5 mg O_2_/L  Intermittent aeration | 6.5 mg NH_4_^+^-N/L,  0.3 mg NO_2_^-^-N/L,  19.1 mg NO_3_^-^-N/L | ~45% | Trojanowicz et al. 2016b |
| Japan | One-stage with anammox carriers | DO: 0.5 mg O_2_/L  Intermittent aeration | ~10 mg TN/L | ~75% | Wu et al. 2021 |
| Switzerland | Two-stage with anammox carriers | Bottom-fed SBRs with anaerobic phases | 0.4 mg NH_4_^+^-N/L,  0.1 mg NO_2_^-^-N/L,  0.9 mg NO_3_^-^-N/L | 90% | Hausherr et al. 2022 |
| Sweden | One-stage with anammox carriers (two reactors connected in sequence) | DO set point: 1.0–2.0 mg L− | 5-15 mg NH_4_^+^-N/L,  0.5 mg NO_2_^-^-N/L,  5-15 mg NO_3_^-^-N/L | < 60% | Gustavsson et al. 2020 |


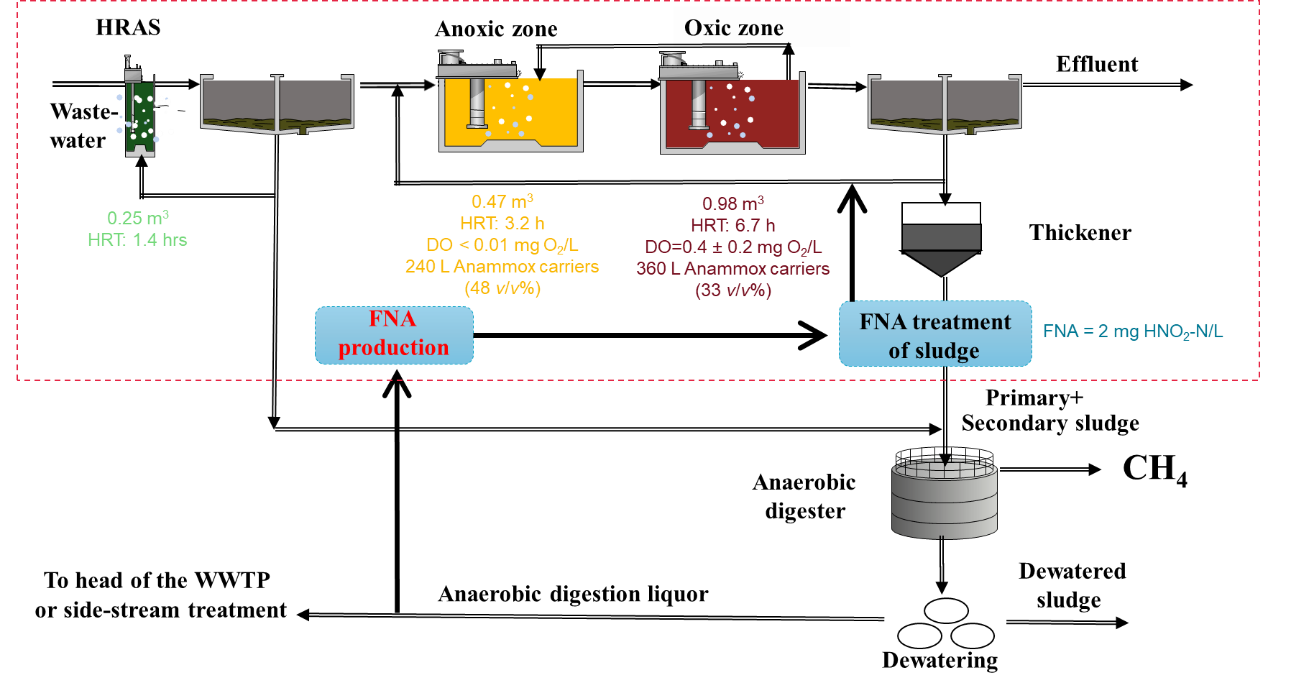


**Fig. S1**. Diagram of the overall process and configuration. Pilot-scale demonstration of the processes was experimentally carried out in the frame of the dashed line.


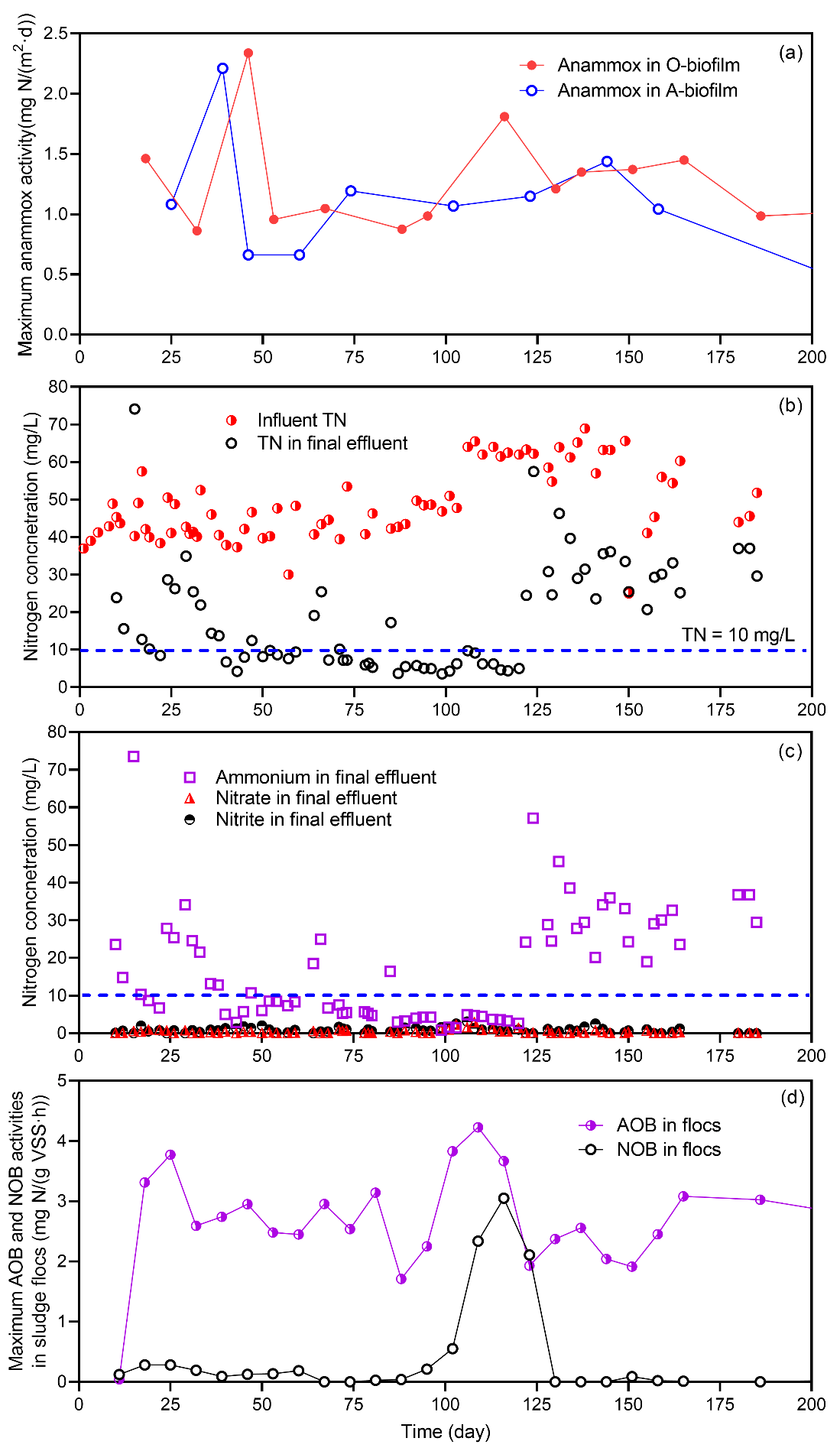


**Fig. S2**. (a) Maximum anammox activity of A-biofilms and O-biofilms. (b) Influent and effluent TN concentrations, and (c) ammonium, nitrite and nitrate concentrations in the effluent. (d) Maximum AOB and NOB activities in sludge flocs.


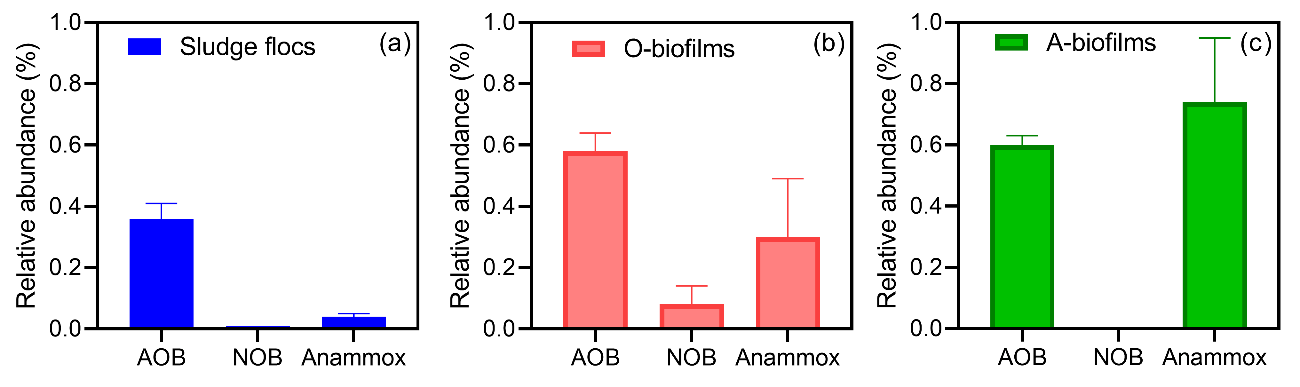


**Fig. S3**. Relative abundances of AOB (Nitrosomonadaceae), NOB (Nitrospira) and anammox bacteria (*Candidatus* Brocadia) in the collected samples of sludge flocs (a), O-biofilms (b) and A-biofilms (c) during the stable operation of the pilot A/O system (on day 450). Note: The abundance of AOB may be overestimated as the Nitrosomonadaceae family may contain genera such as Ellin6067 and mel1-7 that are not belonging to AOB.


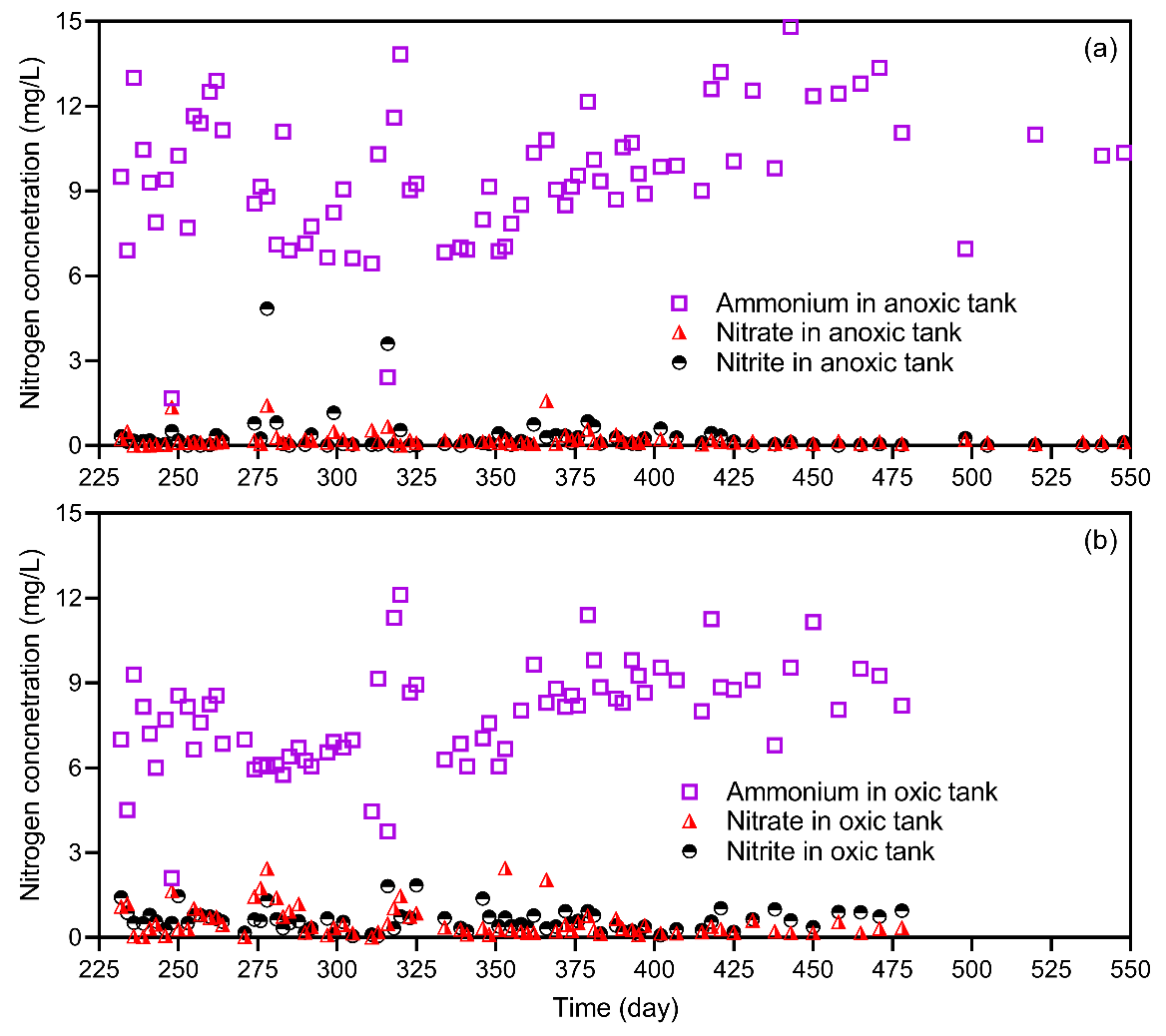


**Fig. S4**. Measured ammonium, nitrite and nitrate concentrations in anoxic tank (a) and oxic tank (b) of the pilot system during the operating period from day 225 to 550.


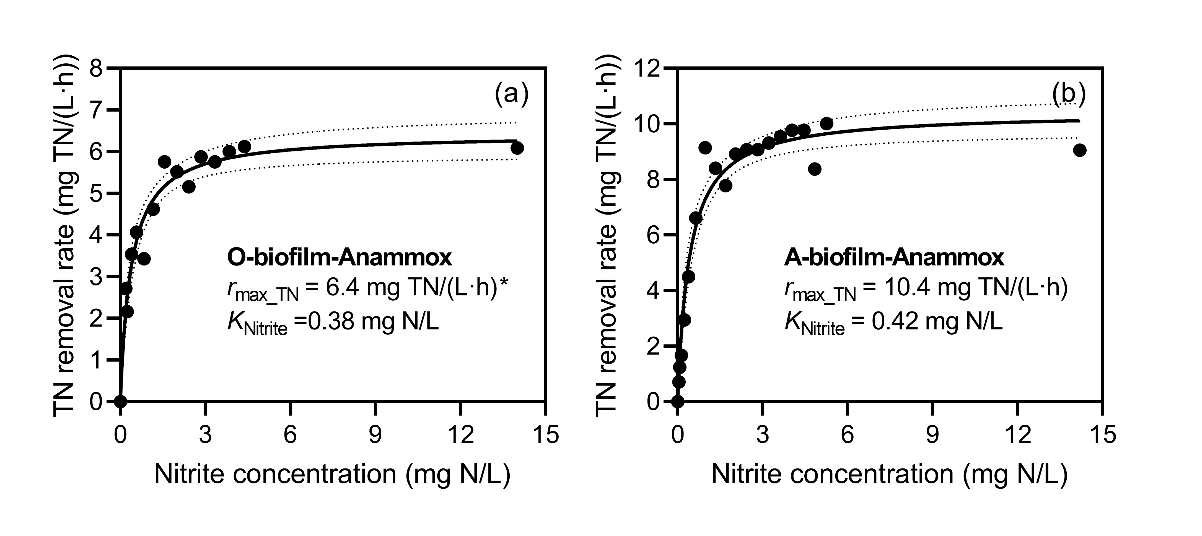


**Fig. S5**. Measured TN removal rates with different nitrite concentrations for (a) O-biofilms tested at DO of 0.4 mg O_2_/L, and (b) A-biofilms anoxically. The measured with data was fitted by using the Monod equation *r* = *r*_max_·(*S*/(*K*+*S*).


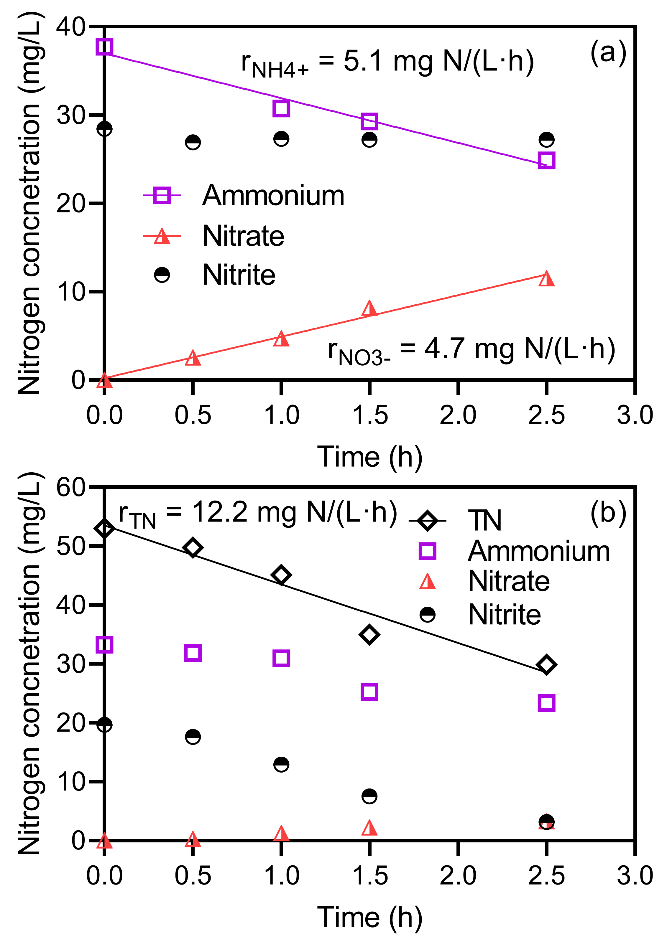


**Fig. S6**. Measured maximum activities of (a) AOB (*r*_NH4+_), NOB (*r*_NO3-_) at DO > 8 mg O_2_/L, and (b) anammox (*r*_TN_) at DO = 0 mg O_2_/L using O-biofilms in the batch tests.
